# Domestic Summary

**Published:** 1839-04-20

**Authors:** 


### DOMESTIC SUMMARY.

*Saratoga Waters.* By N. L. North, M. D.—The subject to which I wish to call the attention of your readers on the present occasion, is the MEDICINAL CHARACTER of the waters of the Saratoga Springs.

By many practitioners these are considered as nothing more nor less than an agreeable and generous laxative, and beneficial in cases of constipation, bilious derangements, dyspepsia, &c. But that there are other qualities that simultaneously co-operate with the aperient effect of the remedy, and are inseparably connected with it, is established beyond all doubt, and should be known by our professsion generally, who are in the habit of sending patients to this village.

There are two methods of testing the nature of an agent that is to be introduced into the materia medica; viz., by carefully watching and recording the *therapeutical effects* of the article by the bedside, and by *chemical analysis*. The latter criterion is good as far as it goes. If sulphur be the only or principal ingredient in a mineral spring, we can bring analogy to decide, *à priori*, what must be the tendency and effects of the water. The same is true of a specimen that is principally saline, or principally chalybeate. We can feel, too, a considerable degree of confidence when two or more constituents are found in combination, provided we are acquainted with the modus operandi of these substances when administered by themselves. But every medical man knows there are limits to this mode of reasoning. How often has the practitioner been surprised at the augmented, diminished, or less irritating effects of some off-hand combination, that would be wholly unlooked for by estimating the separate agency of each article.

So of mineral waters. Chemical analysis can soon decide the exact medicinal nature of a new spring, independently of clinical observation. There are three reasons for this. 1st. Many medicines, such as oxyd of iron, carbonate of iron, pulverized cinchona, calomel, the extracts, the gum-resins when given in pills, capsicum, ginger, &c. pass through the alimentary passages with very little diminution from absorption. Mineral waters, on the contrary, as may be inferred from Dr. Beaumont's experiments, are introduced into the mass of blood by the absorbents of the stomach, without any previous deposition or digestion, and thus these mineral agents, which, in their minute proportions, would be very inert in a state of powder, are admitted to the inner coat of all the bloodvessels, and to the minutest branches of the secretory apparatus. How widely different these two modes of operation must be, all can readily understand. 2d. Chemical analysis cannot appreciate the mutual or qualifying effects of the ingredients on each other, as above stated. 3d. In the language of Dr. James Johnson, of London, (Economy of Health, p. 224,) "Mineral waters contain, in all probability, many agents which we cannot imitate by artificial combinations. This is proved by every day's observation. Thus, the saline, aperient, mineral waters, will produce ten times more effect than the identical materials artificially dissolved and mixed. The same is true with respect to the chalybeate springs. A grain of iron in them is more tonic than twenty grains exhibited according to the pharmacopœia." At page 215—"It does not follow, however, that waters contain no active materials, because chemistry is unable to detect them. Powerful agents may be diffused in waters which are incapable of analysis, or destructible by the process employed for that purpose. The only sure test is EXPERIENCE, of their effects on the human body."

With these qualifying remarks on the proper estimate to be put on chemical analysis, I propose to lay before your readers the composition of some of our principal springs; and, 1st, the *Old Congress Spring*, which is in the South part of the village, just east of Broadway. In our wine gallon, or 231 cubic inches, there are of—

Chloride of sodium, (table salt,) 385 grains,
Hydriodate of soda, 3.5,
Bicarbonate of soda, 8.982,
Bicarbonate of magnesia, 95.788,
Carbonate of lime, 98.098,
Carbonate of iron, 5.075,
Silex, 1.5
Hydrobromate of potass, a trace,
Solid contents, 597.963 grains,
Carbonic acid gas, 311 inches,
Atmospheric air, 7,
Gaseous contents, 318 cubic inches.

The analysis of *Putman's New Congress Spring*, some forty rods north of the old Congress, and on the east of Broadway, has never, to my knowledge, been completed. It is a finely flavoured water, and has many properties similar to those of the Old Congress. This spring alone would do much towards continuing the present immense resort to this village.

Besides these, there is a spring on the premises of Judge Walton, in the north part of the village, which has lately been newly dug and newly curbed, by which process the fresh water has been effectually shut off from the mineral, and it is now considered both by villagers and strangers as a new fountain. It is, indeed, a beautiful water. Possessing the general properties of the other springs already mentioned, it seems to have two peculiarities, viz., the abundance and fixedness of its carbonic acid gas, and its containing only 1-4th or 1-5th the amount of iron there is in the Old Congress. Numerous globules of the gas are seen from a distance floating in the water, and every few seconds explosions of very large bubbles occur at the surface. It bids fair to be an excellent article for bottling, and it has fallen into the hands of gentlemen who, I believe, design to give it circulation. The results of an analysis which are now before me, and which was made the past winter in Albany by Professor Emmons, I would insert, but the proprietors will probably have the process repeated before giving it to the public.

This fountain has attracted great notice during the winter, and has been proved by numbers to possess the usual aperient and diuretic qualities of the other springs above mentioned. Its reconstruction, it is believed, will add greatly to the medicinal resources and permanent prosperity of the village. I need not stay to particularize the other springs, as the above received the principal attention from visitants. The chalybeate springs, of which there are several, are more tonic and less aperient than those already described.

The length to which I have extended this paper forbids my enlarging. The above statements would show that these waters are thoroughly aperient, diuretic and deobstruent. They are also decidedly *tonic*. This fact is fully confirmed by clinical observation. The late Dr. Steel, and all who have practised in this vicinity, as far as my knowledge extends, have found the same tonic effects to accompany the use of the waters. And it is remarkable that this is found to be the case with all the mineral waters of Europe that are not simply sulphureous. The Buxton waters in England, which contain only 15 grains of saline matter in a gallon, and 6 inches of gaseous products, have been found, from a record of 14,906 patients, to be highly stimulating and tonic. The bracing effects of the waters have proved a source of constant embarrassment to Dr. Robertson, of the place, and required continual counteraction. The same report comes from numerous other fountains in Europe; and confirms me in the position that the springs of Saratoga are not only diuretic, aperient and deobstruent, but *tonic*.—*Boston Med. and Surg. Journal.*

